# Mixed culture biocatalytic production of the high-value biochemical 7-methylxanthine

**DOI:** 10.1186/s13036-022-00316-6

**Published:** 2023-01-10

**Authors:** Meredith B. Mock, Ryan M. Summers

**Affiliations:** Department of Chemical and Biological Engineering, The University of Alabama, 35487 Tuscaloosa, AL USA

**Keywords:** 7-methylxanthine, Caffeine, Biocatalysis, *N*-demethylase, Mixed-culture

## Abstract

**Background:**

7-Methylxanthine, a derivative of caffeine noted for its lack of toxicity and ability to treat and even prevent myopia progression, is a high-value biochemical with limited natural availability. Attempts to produce 7-methylxanthine through purely chemical methods of synthesis are faced with complicated chemical processes and/or the requirement of a variety of hazardous chemicals, resulting in low yields and racemic mixtures of products. In recent years, we have developed engineered microbial cells to produce several methylxanthines, including 3-methylxanthine, theobromine, and paraxanthine. The purpose of this study is to establish a more efficient biosynthetic process for the production of 7-methylxanthine from caffeine.

**Results:**

Here, we describe the use of a mixed-culture system composed of *Escherichia coli* strains engineered as caffeine and theobromine “specialist” cells. Optimal reaction conditions for the maximal conversion of caffeine to 7-methylxanthine were determined to be equal concentrations of caffeine and theobromine specialist cells at an optical density (600 nm) of 50 reacted with 2.5 mM caffeine for 5 h. When scaled-up to 560 mL, the simple biocatalytic reaction produced 183.81 mg 7-methylxanthine from 238.38 mg caffeine under ambient conditions, an 85.6% molar conversion. Following HPLC purification and solvent evaporation, 153.3 mg of dried 7-methylxanthine powder was collected, resulting in an 83.4% product recovery.

**Conclusion:**

We present the first report of a biocatalytic process designed specifically for the production and purification of the high-value biochemical 7-methylxanthine from caffeine using a mixed culture of *E. coli* strains. This process constitutes the most efficient method for the production of 7-methylxanthine from caffeine to date.

**Supplementary Information:**

The online version contains supplementary material available at 10.1186/s13036-022-00316-6.

## Background

7-Methylxanthine is a rare compound that is not readily found in nature, except as an intermediate of caffeine biosynthesis in plants [1]. Caffeine (1,3,7-trimethylxanthine) derivatives like 7-methylxanthine are often noted for their ability to cross the blood-brain barrier and act as adenosine receptor antagonists [2], making them attractive as scaffolds for the synthesis of more complex compounds with more finely tuned medical applications [3]. For example, N-heterocyclic carbenes have been constructed from a variety of methylxanthines and have been reported to demonstrate selective toxicity towards certain cancer cell lines and not towards healthy cells [4–7]. Additionally, two specific 7-methylxanthine derivatives clearly demonstrate the diversity and tunability achievable through the use of methylxanthines as scaffolds. KF17837 ((E)-8-(3,4-dimethoxystyryl)-1,3-dipropyl-7-methylxanthine) was designed as a potent adenosine receptor antagonist highly specific for the A2 adenosine receptor [8], which could have applications in areas such as Parkinson’s disease treatment [9]. In contrast, 1,3-dipropyl-7-methylxanthine was designed to sensitize lung carcinoma cells to radiation treatments by inducing apoptotic responses and modifying checkpoints within the cell cycle [10]. Investigations into the medical applications of 7-methylxanthine strongly suggest that the compound can be used to treat and slow the progression of myopia, or nearsightedness, and even prevent its formation. Studies supporting these findings have been conducted in rabbits [11], guinea pigs [12], rhesus monkeys [2], and human children [13]. Additional studies have concluded that 7-methylxanthine is safe for long-term oral administration [14, 15].

**Fig. 1:**
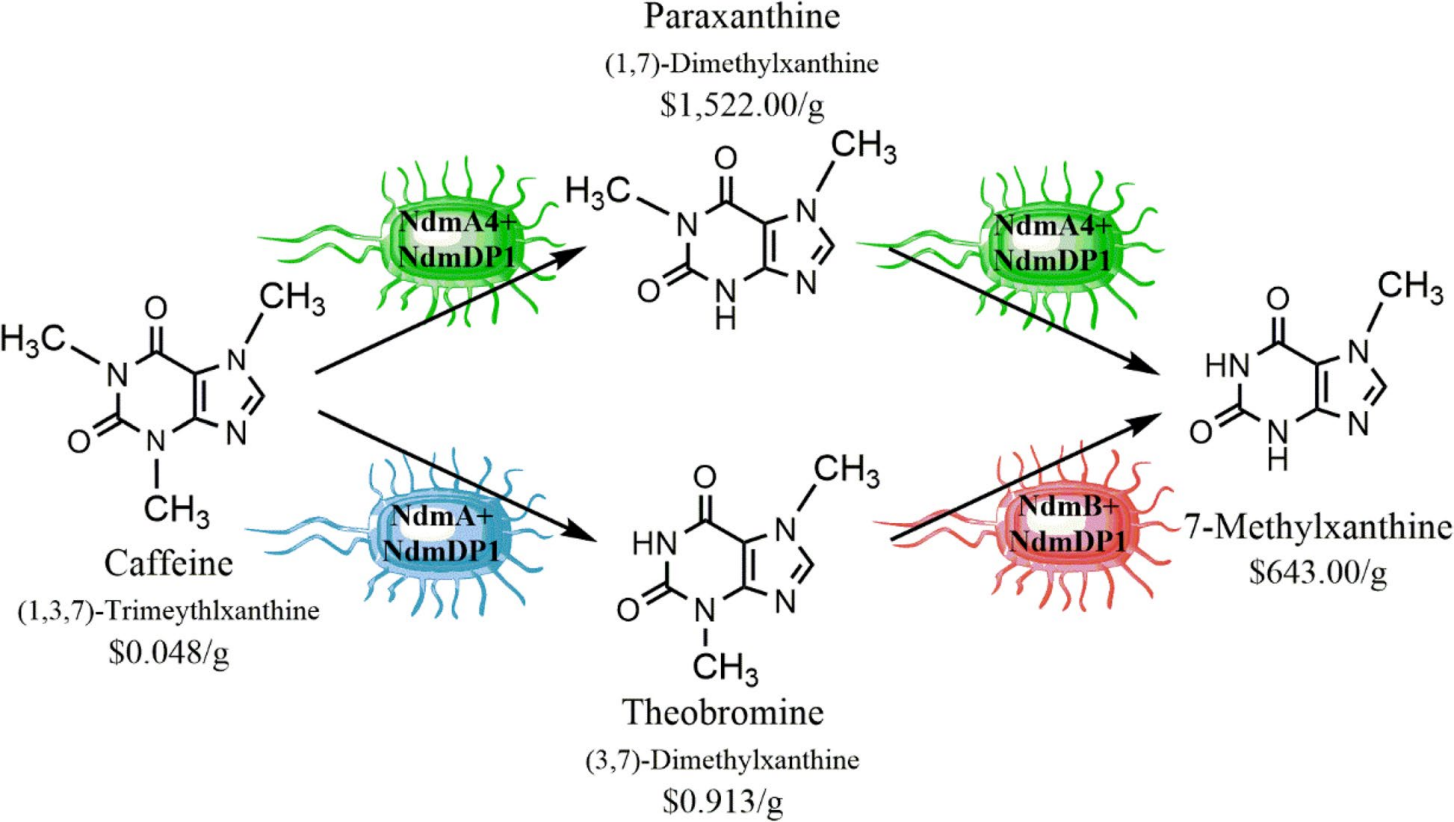
Conversion of caffeine to 7-methylxanthine occurs via theobromine when *N*-demethylation is carried out sequentially by cells expressing the wild-type *ndmA* (blue) and *ndmB* (red) genes, and by paraxanthine when *N*-demethylation is carried out by *E. coli* strain MBM019 expressing the mutant *ndmA4* gene (green). Price of methylxanthines per gram shown is based on lowest retail values from Sigma-Aldrich as of August 2022

Purely chemical methods of 7-methylxanthine synthesis that have attempted to overcome the lack of natural availability are faced with complicated chemical processes and/or the requirement of a variety of hazardous chemicals, such as tetrahydrofuran (THF) and N,N-dimethylformamide (DMF) [16–19]. Attempts at direct N-substitution of xanthine are further complicated by a lack of selectivity resulting from a high similarity in acidity of N_3_-H and N_7_-H, closely followed by N_1_-H. Substitution of the N-H groups therefore follows the expected pattern of N_3_ ≥ N_7_ > N_1_ as indicated by the acidity [20]. Traube purine synthesis, a method considered to be the classical technique for chemically synthesizing substituted purines, is a lengthy process constrained by harsh conditions and poor specificity, even with recent updates and modifications [20–22]. With so many obstacles to the chemical synthesis of 7-methylxanthine, a biosynthetic route offers an alternative method that is simple, safe, reliable, and cost-effective. The retail price of pure 7-methylxanthine can exceed 10,000 times the cost of caffeine (Fig. 1), thus providing an economic incentive to produce 7-methylxanthine from caffeine.

We have characterized a family of five *N*-demethylase enzymes, NdmABCDE, which are able to metabolize caffeine to xanthine in *Pseudomonas putida* CBB5 [23–26]. Initial enzyme characterization conducted in vitro revealed that NdmA is responsible for *N*_*1*_-demethylation of caffeine to theobromine, NdmB carries out the *N*_*3*_-demethylation of theobromine to 7-methylxanthine, and NdmCDE form a complex for *N*_*7*_-demethylation of 7-methylxanthine to xanthine [23]. Within the NdmCDE complex, NdmC was specifically identified as responsible for *N*_*7*_-demethylation. NdmE plays a role as a structural support and is non-catalytic [24]. NdmD is a reductase that is highly specific to the Ndm enzymes and is absolutely required for the biocatalysis of the *N*-demethylation reactions by transferring electrons to NdmA, NdmB, and NdmC.

In the past few years, several metabolically engineered *Escherichia coli* strains have been constructed harboring various combinations of the *ndmABD* genes for the purpose of selective methylxanthine production. For example, 3-methylxanthine has been produced from theophylline (1,3-dimethylxanthine) using *E. coli* strain pDdA [27], theobromine (3,7-dimethylxanthine) has been produced from caffeine using *E. coli* strain pAD1dDD [28], and 7-methylxanthine has been produced from theobromine using *E. coli* strain pBD2dDB [29]. We recently generated a mutant of NdmA, known as NdmA4, that is capable of carrying out *N*_3_-demethylation of caffeine to generate paraxanthine (1,7-dimethylxanthine) as the primary metabolite [30, 31] while also retaining *N*_1_-demethylation activity toward paraxanthine (Fig. 1) [32]. Genetic strain optimization resulted in the creation of *E. coli* strain MBM019 utilizing simultaneous expression of *ndmA4* and *ndmDP1*, an N-terminally truncated version of the NdmD reductase (Fig. S1). Using strain MBM019, we have established optimized processes for the biocatalytic production and purification of paraxanthine and 7-methylxanthine from caffeine [31, 32]. However, these processes are limited by the low reaction rate of the NdmA4 mutant enzyme and result in low yields over longer time periods when compared to processes using wild-type enzymes [27–29].

One potential approach to improve production efficiency of whole-cell catalyzed bioprocesses is the use the of mixed bacterial cultures. *Bacillus* sp. and *Brevumdimonas* sp. have been used successfully in mixed-culture fermentation to generate hydrogen gas from untreated starch powder [33]. The two bacterial strains are more efficient hydrogen producers when operating together than either strain is individually, but increasing the initial substrate concentration beyond 10 g/L had a significant negative impact on hydrogen production. Other systems have reported similar positive results from using a mixed-culture method for recombinant protein expression and product generation, such as the production of limonene by two distinct recombinant *E. coli* strains [34], the production of xylitol by a wild-type *Gluconobacter oxydans* strain and a recombinant *E. coli* BL21 strain [35], the production of methane gas from polyhydroxybutyrate by activated sludge [36], the production of poly- β-hydroxybutyrate by *Bacillus firmus* NII 0830 and *Lactobacillus delbrueckii* NII 0925 [37], and the fermentation of glucose by activated sludge [38]. This compartmentalization of protein expression between two or more hosts can be referred to as “division of labor”, and it can be beneficial in reducing the metabolic burden of an individual cell through increased modularity, as well as by separating incompatible biochemical functions [39].

Here, we demonstrate an optimized mixed-culture microbial platform for the production of 7-methylxanthine from caffeine that is more efficient than previously described methods and generates minimal quantities of side products. This platform uses a mixed culture of *E. coli* strains expressing either *ndmA* or *ndmB* in conjunction with *ndmDP1*, hereafter referred to as pADP1 cells and pBDP1 cells, respectively, thus harnessing the abilities of NdmA and NdmB to jointly convert caffeine to 7-methylxanthine using theobromine as an intermediate. The mixed-culture process constitutes a marketable improvement in conversion efficiency of caffeine to 7-methylxanthine from our previously described method using four rounds of reaction with cells expressing *ndmA4* [31].

## Results

### Strain development and screening

Previous work demonstrated that cells expressing *ndmD* along with either *ndmA* or *ndmB* were able to rapidly consume caffeine or theobromine to produce theobromine or 7-methylxanthine, respectively [26, 28, 29]. This suggested to us that cells containing the wild-type enzymes, which produce theobromine as an intermediate to 7-methylxanthine instead of the paraxanthine generated by the mutant *ndmA4* in strain MBM019, would provide a higher reaction rate and 7-methylxanthine yield. We first attempted to co-express the *ndmAB N*-demethylase genes at different copy numbers along with the *ndmD* or *ndmDP1* reductase gene in a single *E. coli* strain. However, conversion of caffeine to 7-methylxanthine by resting cells stalled out after one hour and totaled less than 25% for each strain assayed (Fig. S2). This reduced activity when both *ndmA* and *ndmB* were co-expressed in a single strain was unexpected, particularly because the two form a stable heterohexameric complex [30], and caused us to shift our investigation to use of a mixed-culture system.

For our mixed-culture system, we constructed caffeine and theobromine “specialist” cells. The caffeine specialist cells contain plasmid pADP1, expressing the *ndmA* and *ndmDP1* genes under control of a single T7 promoter with a synthetic ribosomal binding site between the two genes (Fig. S3). We replaced *ndmA* in pADP1 with *ndmB* to create plasmid pBDP1, which was used for the theobromine specialist cells. We chose the *ndmDP1* truncated reductase gene in place of the full-length *ndmD* gene because cells expressing the truncated gene exhibit higher activity than cells with the full-length gene [31].

### 7-methylxanthine production and reaction condition optimization

To maximize the production of 7-methylxanthine from a mixed culture, we first optimized the ratio of caffeine specialist cells to theobromine specialist cells in the reaction. A resting cell reaction was conducted to compare the efficiency of 7-methylxanthine production from mixed-culture ratios of 100% pADP1 cells to 0% pBDP1 cells (100 A:0B), 75A:25B, 50A:50B, 25A:75B, and 0A:100B. The reactions were carried out in triplicate in 2 mL volumes with a starting caffeine concentration of 1 mM and at an overall total cell OD_600_ of 5. The outcome of these reactions after 5 h is summarized in Table 1. As expected, minimal 7-methylxanthine was generated from the 100A:0B and 0A:100B ratios, with the products that were formed resulting from slight enzyme promiscuity [40]. The 75A:25B ratio consumed most of the caffeine but over half of the theobromine formed was still unreacted, indicating that more pBDP1 cells were required to complete the 7-methylxanthine production. In contrast, the 25A:75B ratio consumed all theobromine generated to produce 7-methylxanthine, but caffeine consumption was reduced due to a lower amount of caffeine specialist cells. The 50A:50B cell ratio generated the highest concentration of 7-methylxanthine from caffeine at 667 ± 19 µM 7-methylxanthine and was selected for future experiments.

**Table 1:** Comparison of Concentrations of Caffeine Consumed and Compounds Produced by Varied Cell Densities of pADP1 and pBDP1^a^

OD_600_ (NdmA/NdmB)	Caffeine Consumed (μM)	Theobromine Produced (μM)	7-Methylxanthine Produced (μM)
100:0	1022 ± 19	988 ± 24	10 ± 5
75:25	961 ± 64	545 ± 17	315 ± 27
50:50	643 ± 25	24 ± 11	667 ± 19
25:75	493 ± 43	-	481 ± 48
0:100	62 ± 57	-	22 ± 2

^a^Reported concentrations are averages of triplicate 1 mL reactions sampled at the conclusion of a 5-hour reaction initiated by the addition of 1 mM caffeine

**Fig. 2:**
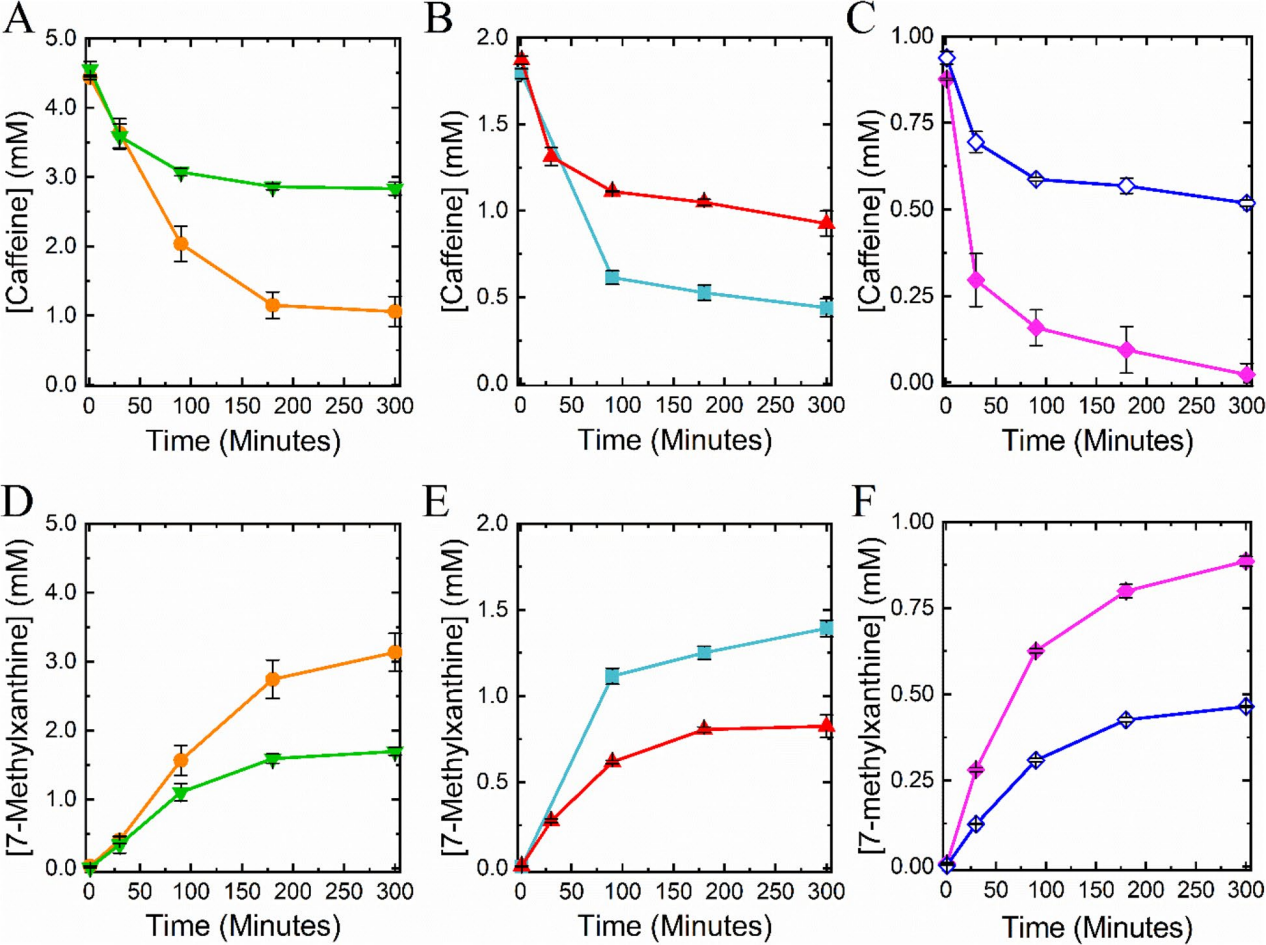
Mixed-culture resting cell reactions consumed caffeine and generated 7-methylxanthine. Equal concentrations of pADP1 and pBDP1 cells were reacted with the following initial caffeine concentrations and at the following optical densities: **A**, **D** 5 mM caffeine, OD_600, total_ = 50 (●, orange) & OD_600, total_ = 25 (▼, green), **B**, **E** 2 mM caffeine, OD_600, total_ = 20 (■, teal) & OD_600, total_ = 10 (▲, red), **C**, **F** 1 mM caffeine, OD_600, total_ = 10 (♦, pink) & OD_600, total_ = 5 (◊, blue). **A**-**C** represent the caffeine consumed, and **D**-**F** represent the 7-methylxanthine generated. All reactions were conducted in triplicate, and the representative data points are averages of the concentrations at a given time point with the corresponding standard deviation

After optimizing the ratio of pADP1 cells to pBDP1 cells, we varied cell density (total OD_600_ of 5, 10, 20, 25, and 50) and substrate concentration (1, 2, and 5 mM) to determine ideal reaction conditions (Fig. 2, Table S1). As expected, in all cases the higher cell density was more efficient at both consuming caffeine and generating 7-methylxanthine, and 7-methylxanthine concentrations nearly doubled with doubling cell concentrations (Fig. 2D-F). Furthermore, theobromine concentrations were minimal at the end of all reactions. The only reaction to show complete consumption of caffeine contained 1 mM caffeine with an OD_600_ of 10, which produced 886 ± 14 µM 7-methylxanthine (Fig. 2C and F). We also examined caffeine concentrations of 10mM, 25mM, and 50 mM with a total OD_600_ of 100, but we observed a decreasing activity with increased caffeine concentrations, suggesting that high caffeine concentrations may be inhibiting to the reaction (data not shown).

**Fig. 3:**
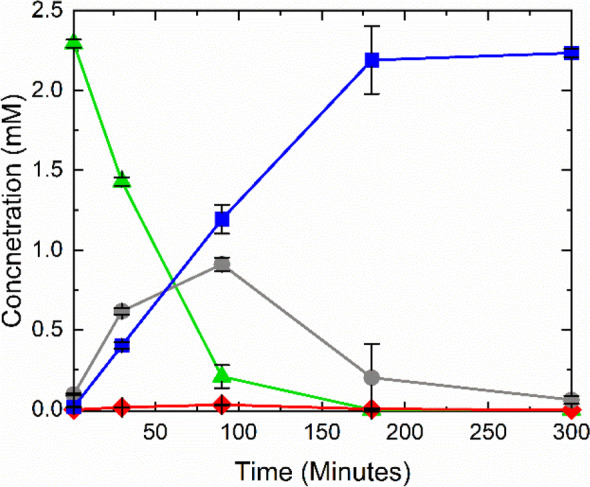
Equal mixtures of pADP1 and pBDP1 cells at a total OD_600_ of 50 were reacted with 2.5 mM caffeine and the resulting reaction products were quantified over time. Caffeine (▲, green) was initially converted primarily to theobromine (●, grey) and then to 7-methylxanthine (■, blue) with only minor production of paraxanthine (♦, red). All reactions were conducted in triplicate, and the representative data points are averages of the concentrations at a given time point with the corresponding standard deviation

A total cell OD_600_ of 50 was selected as the optimal cellular concentration for the production of 7-methylxanthine from caffeine. To achieve complete consumption of caffeine, cells at the selected density were reacted with approximately 2.5 mM caffeine (Fig. 3), rather than the previous 5 mM (Fig. 2A and D), resulting in the complete conversion of 2,293 ± 24 µM caffeine to 61 ± 25 µM theobromine and 2,233 ± 26 µM 7-methylxanthine after five hours. During the reaction caffeine was completely consumed within three hours, but it took five hours to convert most of the theobromine to 7-methylxanthine.

### 7-methylxanthine purification and recovery

The 2,233 ± 26 µM 7-methylxanthine produced in our small-scale mixed resting cell assay suggested that there would be enough product for isolation given a larger reaction volume. Therefore, we proceeded to scale up the reaction. The pADP1 and pBDP1 *E. coli* strains were each grown in three 2.8 L Fernbach flasks for a total of six 1 L cultures, resulting in sufficient cells for a 560 mL mixed resting cell reaction containing a 50A:50B cell ratio (total OD_600_ of 50) and 2.5 mM caffeine. The cell-caffeine mixture was allowed to react for five hours to ensure maximum conversion before harvesting. At the conclusion of the large-scale reaction, caffeine was fully degraded, producing 2.14 mM 7-methylxanthine and 0.235 mM theobromine (Fig. S4). This resulted in an 85.6 mol% conversion of caffeine to 7-methylxanthine and a 9.4 mol% conversion to theobromine. The remaining 5 mol% of consumed caffeine could be accounted for in two unidentified HPLC peaks at 3.44 and 4.58 min not observed in a cell-only control [31].

Preparation of the reaction by 0.2 μm filtration and the addition of methanol to a final concentration of 5% for HPLC purification resulted in 542.85 mL supernatant, which was separated by preparatory-scale HPLC as described previously [31, 32]. Given that the final concentration of 7-methylxanthine produced was 2.14 mM, the theoretical maximum amount of 7-methylxanthine that could be recovered from this process was 183.8 mg. The purification process was successful in separating contaminants and unwanted compounds from the desired 7-methylxanthine with minimal loss of the product, resulting in a separation efficiency of 93.30% (Table S2). Following purification, the collected product was dried to a powder, allowing for the recovery of 153.3 mg 7-methylxanthine (Fig. S5). Comparing the theoretical mass of 7-methylxanthine in the reaction buffer to the actual collected mass, this process allowed for a recovery of 83.4 wt%. Thus, combination of the reaction and purification processes described here could result in production of 328.2 mg 7-methylxanthine per liter of mixed-culture resting cell reaction.

### Analytical characterization of 7-methylxanthine

7-Methylxanthine purity was analyzed using authentic HPLC standards and the retention times were confirmed to be the same (Fig. S5). ^1^ H-NMR was also used to confirm the identity of the biologically produced 7-methylxanthine (Fig. S6). The presence of peaks was confirmed at δ 11.45 (1 H) and 10.83 (1 H) corresponding to the two –NH groups, δ 7.88 (1 H) corresponding to –N=CH, and δ 3.86 (3 H) corresponding to the –CH_3_ group. The peaks at δ 3.33 and δ 2.51 have been confirmed to correspond to water and DMSO, respectively.

## Discussion

7-Methylxanthine has previously been produced from theobromine by a strain of *E. coli* expressing combinations of NdmB and NdmD [29]. However, caffeine is less expensive and more abundant than theobromine, making caffeine a preferred substrate for the biocatalytic production of 7-methylxanthine. Recently, we reported on our ability to synthesize 7-methylxanthine from caffeine via paraxanthine using a mutant *N*-demethylase, NdmA4 [31]. However, we were only able to demonstrate complete conversion of 5 mM caffeine to 7-methylxanthine after four rounds of reaction, each time supplemented with fresh cells [32]. For the present work, we theorized that production of 7-methylxanthine from caffeine would be improved by using the wild-type *ndm* genes operating in tandem to convert caffeine first to theobromine instead of paraxanthine, specifically expressing *ndmA* and *ndmB* in individual cell cultures and combining the cultures together in one reaction. NdmA and NdmB have been demonstrated individually to be highly efficient in their ability to degrade caffeine to theobromine and theobromine to 7-methylxanthine, respectively (Fig. S2). Cells containing NdmA have been shown to degrade 1 mM caffeine to theobromine within 90 min [28, 31], and NdmB cells can degrade 0.5 mM theobromine to 7-methylxanthine in one hour [29]. These rates suggest a greater caffeine to 7-methylxanthine conversion efficiency is possible using NdmA and NdmB over the mutant NdmA4.

While we elected to use the wild type *ndmA* and *ndmB* genes, a truncated version of *ndmD* which promotes a higher catalytic activity [31], known as *ndmDP1* (Fig. S1), was used in preference to the full-length reductase. Furthermore, coexpression of *ndmDP1* and *ndmA* separate from *ndmDP1* and *ndmB* improves the ratio of reductase to *N*-demethylase within each cell, ensuring that NdmDP1 does not become the limiting factor with NdmA and NdmB competing for access to the reductase. Our mixed-culture method also provides advantages regarding the regulation and control of protein concentrations and ratios. We cloned the gene combinations into the same expression vector at one copy per gene and under the control of the same promoter, thereby ensuring that there would be approximately the same number of plasmids per cell and roughly the same gene expression rate. The two strains, pADP1 and pBDP1, could then be grown and protein expressed separately so that the concentration of NdmA to NdmB within each reaction could be varied by simply adjusting the quantity of cells from each strain. A comparison of 7-methylxanthine production by strain can be found in Table 2.

**Table 2:** Comparison of 7-Methylxanthine Production by Various Engineered *E. coli* Strains^a^

Strains	Substrate Consumed	7-Methylxanthine Produced	Rate	Reference
pBD2dDB	0.5 mM Theobromine	0.5 mM	0.25 mmol/L•hr	[29]
MBM019	4.33 mM Caffeine	2.12 mM	0.424 mmol/L•hr	[32]
pADP1 & pBDP1	2.5 mM Caffeine	2.23 mM	0.743 mmol/L•hr	This study

^a^All plasmids were expressed in *E. coli* BL21(DE3)

During our previous optimization of paraxanthine production, we noted that higher concentrations of cells gave a greater conversion efficiency, but also started exhibiting a secondary degradation step resulting in an additional product (7-methylxanthine) at the expense of our compound of interest [31]. We were further able to produce 7-methylxanthine using *E. coli* strain MBM019, but the process was very time- and labor-intensive, requiring four rounds of cell growth and resting cell reactions [32]. For this reason, we tested a range of cell densities from the combination of pADP1 and pBDP1 cells to determine the optimal total cell concentration for the production of 7-methylxanthine from caffeine that would most effectively minimize any side products or additional degradation. We also wanted to determine the optimized conditions for complete conversion of caffeine as total substrate conversion would reflect high reaction efficiency, and consumption of the entire substrate would improve purification. From these parameters, we were able to determine that a 1:1 mixture of pADP1 and pBDP1 cells at an overall OD_600_ of 50 was most effective for the complete degradation of 2.5 mM caffeine to 7-methylxanthine as the primary product in less than 5 h. Additionally, Fig. 3 clearly demonstrates the simultaneous reactions occurring within the mixed-culture system, where caffeine is first being converted to theobromine which is then rapidly converted to 7-methylxanthine. By utilizing both HPLC and NMR techniques to confirm the identity of the produced compound, we have verified that a mixed culture of *ndmA* and *ndmB* expressing cells can produce 7-methylxanthine as the primary product from caffeine via theobromine.

Whole-cell biosynthetic production of methylxanthines offers an alternative to purely synthetic routes. These synthetic pathways frequently require multiple steps, strong solvents, high temperatures, high pressures, long reaction times, result in a mixture of compounds, and can require the use of catalysts [20–22, 41, 42]. One potential use for this technology is the generation of value-added products from waste. Waste from coffee processing plants, which would make an excellent substrate for this type of process, only has residual amounts of caffeine. Specifically, spent coffee grounds when extracted have previously been demonstrated to yield 1.1–2.5 mM residual caffeine, concentrations that fit well with the process that we have established [43]. The biosynthetic production of 7-methylxanthine from caffeine utilizes a low-cost, easily accessible substrate and the use of engineered *E. coli* as a biocatalyst offers an economically and environmentally friendly system for efficient and highly specific production of 7-methylxanthine. In addition, the rate at which caffeine is consumed globally in the form of tea and coffee has led to the generation of a significant quantity of caffeine-rich waste that could be harnessed through these novel biosynthetic pathways, keeping this residual caffeine out of the soil and ground water.

Due to the low cost of purified caffeine, production of 7-methylxanthine from concentrations of caffeine higher than what can be found in waste sources is an attractive goal. However, the observed decrease in resting cell activity with increasing caffeine concentrations limits the ability to use our current system at high substrate concentrations for gram-scale production of methylxanthines. Fortunately, there are numerous routes of optimization that might prove beneficial in overcoming this problem, depending on the underlying issue. Mutagenesis of the *N*-demethylase genes may relieve the apparent substrate inhibition, allowing use of higher caffeine concentrations. Other genetic optimizations, such as increasing the enzyme levels in the cells may also help to increase the rate at which the substrate is consumed without need for additional cells. Currently, the resting cell reactions are separated from the cell growth and protein production due to differences in required temperatures for gene expression and biotransformations. Expression of the *N*-demethylase genes in a different host, such as *Pseudomonas putida*, may improve soluble enzyme production, and may allow the reaction to occur while cells are growing. Process optimization techniques that may serve to enhance 7-methylxanthine production include the use of a fed-batch method [44, 45] or cell immobilization [46–48]. Thus, while this study demonstrates the first mixed-cell production of methylxanthines, additional genetic and process optimizations will be required to improve substrate consumption and product yield.

## Conclusion

We have presented the first report of a biocatalytic process designed specifically for the production and purification of the high-value biochemical 7-methylxanthine from caffeine using a mixed culture of *E. coli* strains. The process described here produced 183.81 mg 7-methylxanthine from 238.38 mg caffeine under ambient conditions using a simple biocatalytic reaction prior to further purification steps. Of the original 238.38 mg of caffeine, 21.89 mg is accounted for as unconverted theobromine and an estimated 31 mg was lost as removed methyl groups giving an estimated 99.09% conversion efficiency. We further isolated and collected 153.3 mg 7-methylxanthine powder via prep-scale HPLC with a purification yield of 83.8%. This process constitutes the most efficient method for the production of 7-methylxanthine from caffeine to date.

## Materials and methods

### Chemicals and reagents

Caffeine was purchased from J.T. Baker (Phillipsberg, NJ, USA). 7-methylxanthine was acquired from Alfa Aesar (Haverhill, MA, USA). Theobromine was bought from Acros Organics (Fair Lawn, NJ, USA). Luria-Bertani media was made in accordance with the protocol described by MacWilliams, et al. [49]. Isopropyl β-D-thiogalactopyranoside (IPTG) was bought from INDOFINE Chemical Company (Hillsborough, NJ, USA). All PCR reactions were performed utilizing Phusion HF polymerase. All PCR reagents and restriction enzymes were obtained from New England BioLabs (Ipswich, MA, USA). Antibiotics were purchased from AMRESCO (Solon, OH, USA). The HPLC-grade methanol used during chromatography separations was from J.T. Baker (Phillipsburg, NJ, USA).

### Plasmid construction

All plasmids used in this study are listed in Table 3, and a list of all primers (Table S3) and their corresponding fragments used for plasmid construction (Table S4) can be found in the Supplementary Information. Plasmids were constructed such that all of the genes are under the control of the T7 promoter, allowing for IPTG-dependent selective induction of expression. Detailed plasmid construction is described in the Supplementary Information. For plasmids pAD3, pBD3, pADP1, and pBDP1, two genes were cloned as a bicistronic insert under control of a T7 promoter with the ribosomal binding site upstream of the first multiple cloning site from pACYCDuet-1 (pETrbs2, GAAGGAGATATACC) placed between the two genes (Fig. S3).

### Strain construction

*E. coli* BL21(DE3) was used as the parent strain to construct both strains used in this research. A complete list of strains with their descriptions is located in Table 3. Plasmids were transformed into chemically competent *E. coli* BL21(DE3) and recombinant strains were plated on LB agar plates [49] containing 100 µg/mL ampicillin.

**Table 3:** Complete List of Plasmids and Strains Used in this Study

Name	Characteristics	Source
**Plasmids**		
pET-32a(+)	Amp^R^, T7 promoter, C-terminal His_6_ tag, pBR322 origin	Novagen
dDA	pACYCDuet-1 with one copy of *ndmD* and one copy of *ndmA*	[27]
dDB	pACYCDuet-1 with one copy of *ndmD* and one copy of *ndmB*	[29]
dAA	pACYCDuet-1 with two copies of *ndmA*	[27]
dBB	pACYCDuet-1 with two copies of *ndmB*	[29]
pAD3	pET-32a(+) with *ndmA* and *ndmD* linked through a pETrbs2	This study
pBD3	pET32a(+) with *ndmB* and *ndmD* linked through pETrbs2	This study
dDP1DP1	pACYCDuet-1 with two copies of *ndmDP1*	This study
pADP1	pET-32a(+) with one copy of *ndmA* and one copy of *ndmDP1*	This study
pBDP1	pET-32a(+) with one copy of *ndmB* and one copy of *ndmDP1*	This study
**Strains**		
*E. coli* BL21(DE3)	F^-^ *ompT hsdS*_*B*_ (r^-^_B_m^-^_B_) *gal dcm* (DE3)	Invitrogen
*E. coli* pADP1	BL21(DE3) pADP1	This study
*E. coli* pBDP1	BL21(DE3) pBDP1	This study
*E. coli* pAD3dDB	BL21(DE3) pAD3 dDB	This study
*E. coli* pAD3dBB	BL21(DE3) pAD3 dBB	This study
*E. coli* pBD3dAA	BL21(DE3) pBD3 dAA	This study
*E. coli* pBD3dDA	BL21(DE3) pBD3 dDA	This study
*E. coli* pADP1dBB	BL21(DE3) pADP1 dBB	This study
*E. coli* pBDP1dAA	BL21(DE3) pBDP1 dAA	This study
*E. coli* pAD3dDD	BL21(DE3) pAD3 dDD	This study
*E. coli* pBD3dDD	BL21(DE3) pBD3 dDD	This study

### Cell growth and protein expression

For all mixed-culture reactions, the two *E. coli* strains were grown separately, and protein was expressed as described by Mock, et al. [40]. Briefly, cells were grown in LB with ampicillin at 37 °C and shaking at 200 rpm. Upon reaching an OD_600_ of ~ 0.5, the cells were supplemented with sterile iron chloride at a final concentration of 10 µM and the culture was shifted to 18 °C. To induce gene expression, IPTG was added to a final concentration of 0.1 mM when the OD_600_ reached 0.8, and the cells were grown post-induction for an additional 14–16 h at 18 °C with 200 rpm shaking. Cells were harvested by centrifugation at 10,000 x *g* for 10 min at 4 °C. Small scale cultures were carried out in 15 mL media. Cultures designated for product isolation were grown in two batches of 1 L of media per strain contained within 2.8-L Fernbach flasks.

### Reaction conditions for 7-methylxanthine production by mixed culture

Harvested cells were washed and resuspended in ice cold 50 mM potassium phosphate (KP_i_) buffer (pH 7.5). Unless otherwise indicated, resting cell assays were conducted at an overall OD_600_ of 5, in test tubes at a volume of 2 mL, and an initial caffeine concentration of 1 mM in KP_i_ buffer. Reactions were carried out at 30 °C and 200 rpm shaking for 5 h, and 100 µL samples were taken periodically for HPLC analysis to determine methylxanthine concentrations using the appropriate standards.

Washing and resuspension of cells designated for the large-scale reaction resulted in 125 mL of NdmA cells at an OD_600_ of 112 and 120 mL of NdmB cells at an OD_600_ of 171. The volume of the large-scale reaction for production and purification of 7-methylxanthine was maximized based on harvested cell density to a total of 560 mL and a caffeine concentration of 5 mM. The overall OD_600_ of 50 consisted of 50% pADP1 cells and 50% pBDP1 cells. The reaction was incubated in a 2.8-L Fernbach flask at 30℃ and 200 rpm shaking for 5 h. At the conclusion of the reaction, the cells were harvested by centrifugation at 10,000 x *g* for 10 min at 4℃ to allow for collection of the supernatant for purification.

### Preparatory HPLC

The harvested supernatant was filtered through a 0.2 μm filter prior to HPLC purification, and the final collected volume of supernatant measured 517 mL. 7-Methylxanthine purification was completed using a ThermoScientific Hypersil BDS C18 preparatory HPLC column (20 mm diameter x 150 mm length), which was connected to a Shimadzu LC-20AT HPLC system equipped with a photodiode array detector to detect and record the UV-visible absorption spectra. A mobile phase of 5:95:0.5 (vol/vol/vol) methanol-water-acetic acid at a flow rate of 2.5 mL/min was used. An isocratic program was developed using two pumps operating at 2.5 mL/min so that one pump would load the post reaction mixture for 4 min (10 mL total) and the second pump would deliver the mobile phase. A total of 25.85 mL of methanol was added to the reaction supernatant to match the HPLC concentration of 5% MeOH to prevent a swing in MeOH concentration from affecting the HPLC chromatograph. The supernatant-methanol mixture was loaded onto the column at a rate of 2.5 mL/min for 4 min, resulting in a total of 10 mL of supernatant loaded each round. After 53 rounds of separation, approximately 1 L volume of 7-methylxanthine solution was collected. The solution was concentrated using a rotary evaporator at 70℃ and 200–220 mbar, reducing the volume to 310 mL. The concentrated solution was finally dried at 140℃ to produce 7-methylxanthine powder (Fig. S5). Supernatant was loaded onto the column in an overlapping pattern such that 7-methylxanthine peaks did not overlap with undesired products but rounds of separation could be run more rapidly.

### Analytical procedures

7-Methylxanthine was identified and quantified using the same HPLC system as described above. A ThermoScientific Hypersil BDS C18 HPLC column (4.6 mm inner diameter x 150 mm length) was used as the stationary phase. A mobile phase of 7.5:92.5:0.5 (vol/vol/vol) methanol-water-acetic acid at a flow rate of 0.5 mL/min. Purity of the 7-methylxanthine was confirmed using HPLC and NMR (Figs. S5 and S6). The NMR results were obtained from the NMR facility in the Chemistry Department of the University of Alabama. The spectrum was recorded in DMSO-*d*_*6*_ with a Bruker DRX 500 NMR spectrometer at 299 K. The chemical shifts were relative to DMSO-*d*_*6*_ using the standard δ notation in parts per million.

## Supplementary Information

**Additional file 1: Figure S1.** Gene maps comparing *ndmD* (green) to the truncated reductase, *ndmDP1* (blue). Regions encoding conserved protein domains are shown above the genes. **Figure S2.** Strain comparison of 7-methylxanthine (red) and theobromine (blue) end-of-reaction production from 1 mM of substrate. Caffeine was used as the substrate for all reactions except for pBD3dDD, which used theobromine. Strains harboring both NdmA and NdmB simultaneously produce less overall product than strains harboring only NdmA or NdmB. *Escherichia coli* BL21(DE3) was used as the host for all strains. Listed below each strain is the hypothetical copy number of each gene, estimating a copy number of 40 for pET28a(+)-based plasmids and 10 for plasmids derived from pACYCDuet-1. Estimated copy numbers were taken from the Novagen Duet Vectors user protocol TB340 Rev. F 0211JN, Table 2 (page 4 of 12). **Figure S3.** Representative gene maps. A) Gene map of pAD3 depicting the T7 promoter (yellow), and NdmA (turquoise) connected to NdmD (green) by a ribosomal binding site, pETrbs2 (pink). A similar construction was used for plasmids pBD3, pADP1, and pBDP1. B) Gene map of dDP1DP1 (blue), with both genes under control of their own T7 promoter. **Figure S4.** HPLC chromatograph of the large-scale reaction supernatant confirming the caffeine metabolites at the conclusion of the large-scale assay for production and separation. TB, theobromine; 7-MX, 7-methylxanthine. Unidentified peaks have previously been attributed to the host strains or potential methyluric acids [9]. **Figure S5.** HPLC chromatograph of the 7-methylxanthine collected from the HPLC separation process. Inset: Purified powdered 7-methylxanthine collected post HPLC purification and solvent evaporation. **Figure S6.** ^1^H-NMR of HPLC-purified and dried 7-methylxanthine in DMSO. **Table S1.** End of Reaction Concentrations for Fig. 2. **Table S2.** Mass of Products Before and After HPLC Purification. Supplemental Methods. **Table S3.** Primers and Templates Used for the Generation of PCR Inserts. **Table S4.** Primers Used in Plasmid Construction.

## Data Availability

All data generated or analyzed during this study are included in this published article [and its supplementary information files].
