# Supplementary material for: Mixed culture biocatalytic production of the high-value biochemical 7-methylxanthine
Source: J Biol Eng. 2023 Jan 10;17:2. doi: 10.1186/s13036-022-00316-6 (PMC9830774; doi:10.1186/s13036-022-00316-6)
Supplement: Supplementary file 1 — Additional file 1: Figure S1. Gene maps comparing ndmD (green) to the truncated reductase, ndmDP1 (blue). Regions encoding conserved protein domains are shown above the genes. Figure S2. Strain comparison of 7-methylxanthine (red) and theobromine (blue) end-of-reaction production from 1 mM of substrate. Caffeine was used as the substrate for all reactions except for pBD3dDD, which used theobromine. Strains harboring both NdmA and NdmB simultaneously produce less overall product than strains harboring only NdmA or NdmB. Escherichia coli BL21(DE3) was used as the host for all strains. Listed below each strain is the hypothetical copy number of each gene, estimating a copy number of 40 for pET28a(+)-based plasmids and 10 for plasmids derived from pACYCDuet-1. Estimated copy numbers were taken from the Novagen Duet Vectors user protocol TB340 Rev. F 0211JN, Table 2 (page 4 of 12). Figure S3. Representative gene maps. A) Gene map of pAD3 depicting the T7 promoter (yellow), and NdmA (turquoise) connected to NdmD (green) by a ribosomal binding site, pETrbs2 (pink). A similar construction was used for plasmids pBD3, pADP1, and pBDP1. B) Gene map of dDP1DP1 (blue), with both genes under control of their own T7 promoter. Figure S4. HPLC chromatograph of the large-scale reaction supernatant confirming the caffeine metabolites at the conclusion of the large-scale assay for production and separation. TB, theobromine; 7-MX, 7-methylxanthine. Unidentified peaks have previously been attributed to the host strains or potential methyluric acids [9]. Figure S5. HPLC chromatograph of the 7-methylxanthine collected from the HPLC separation process. Inset: Purified powdered 7-methylxanthine collected post HPLC purification and solvent evaporation. Figure S6. 1H-NMR of HPLC-purified and dried 7-methylxanthine in DMSO. Table S1. End of Reaction Concentrations for Fig. 2. Table S2. Mass of Products Before and After HPLC Purification. Supplemental Methods. Table S3. Primers and Templates Use [file 13036_2022_316_MOESM1_ESM.docx]

Supplementary information for Mixed Culture Biocatalytic Production of the High-Value Biochemical 7-Methylxanthine

Meredith B. Mock and Ryan M. Summers*

Department of Chemical and Biological Engineering, The University of Alabama, Tuscaloosa, AL 35487, USA

*To whom correspondence should be addressed. Email: rmsummers@eng.ua.edu, Phone: 1-205-348-3169, Fax: 1-205-348-7558


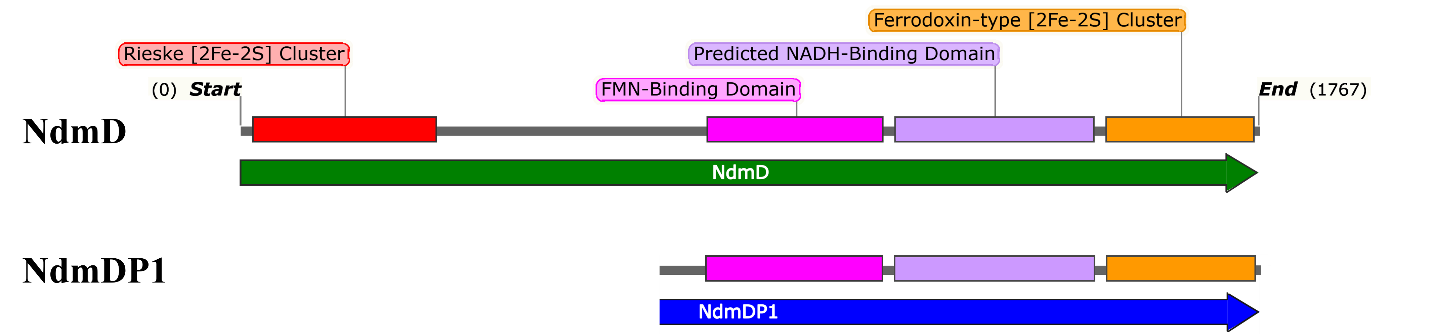


**Figure S1.** Gene maps comparing *ndmD* (green) to the truncated reductase, *ndmDP1* (blue). Regions encoding conserved protein domains are shown above the genes.


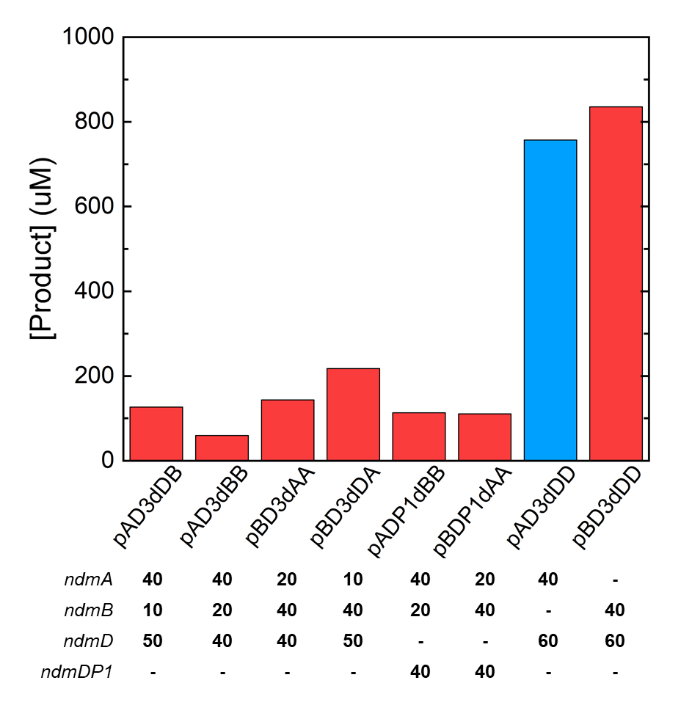


**Figure S2.** Strain comparison of 7-methylxanthine (red) and theobromine (blue) end-of-reaction production from 1 mM of substrate. Caffeine was used as the substrate for all reactions except for pBD3dDD, which used theobromine. Strains harboring both NdmA and NdmB simultaneously produce less overall product than strains harboring only NdmA or NdmB. *Escherichia coli* BL21(DE3) was used as the host for all strains. Listed below each strain is the hypothetical copy number of each gene, estimating a copy number of 40 for pET28a(+)-based plasmids and 10 for plasmids derived from pACYCDuet-1. Estimated copy numbers were taken from the Novagen Duet Vectors user protocol TB340 Rev. F 0211JN, Table 2 (page 4 of 12).

**
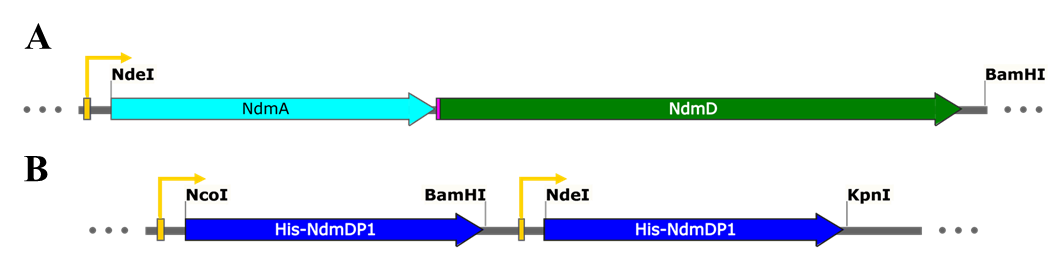
**

**Figure S3.** Representative gene maps. A) Gene map of pAD3 depicting the T7 promoter (yellow), and NdmA (turquoise) connected to NdmD (green) by a ribosomal binding site, pETrbs2 (pink). A similar construction was used for plasmids pBD3, pADP1, and pBDP1. B) Gene map of dDP1DP1 (blue), with both genes under control of their own T7 promoter.


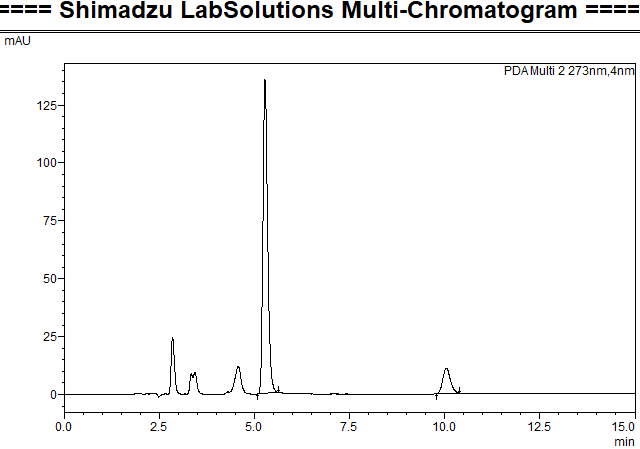


**TB**

**7-MX**

**Figure S4.** HPLC chromatograph of the large-scale reaction supernatant confirming the caffeine metabolites at the conclusion of the large-scale assay for production and separation. TB, theobromine; 7-MX, 7-methylxanthine. Unidentified peaks have previously been attributed to the host strains or potential methyluric acids [9].


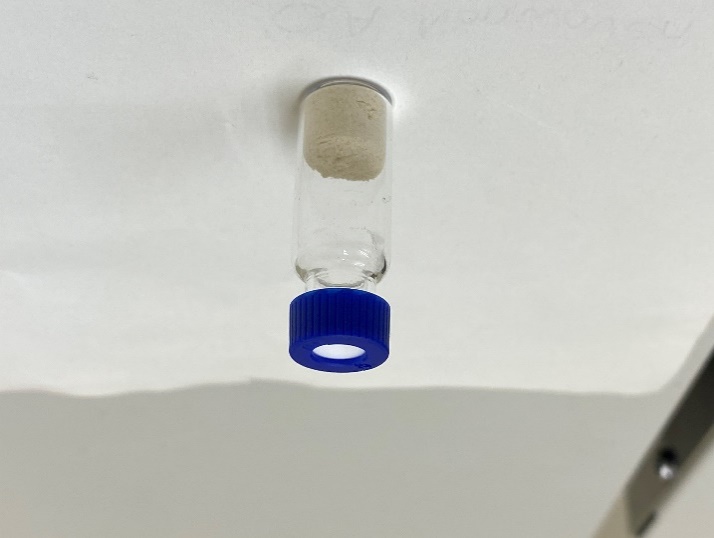

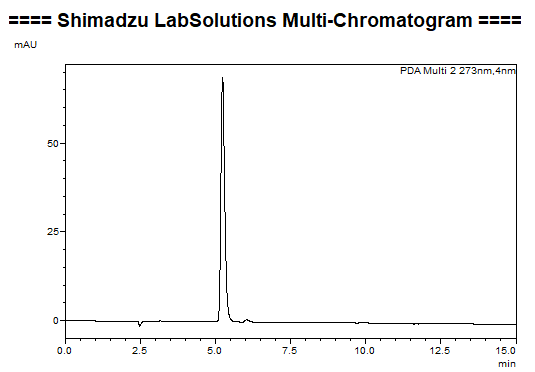


**Figure S5.** HPLC chromatograph of the 7-methylxanthine collected from the HPLC separation process. Inset: Purified powdered 7-methylxanthine collected post HPLC purification and solvent evaporation.


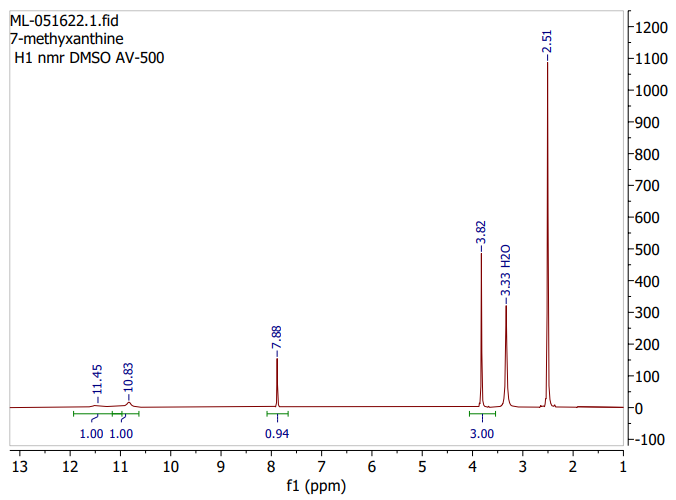


DMSO

**Figure S6.** ^1^H-NMR of HPLC-purified and dried 7-methylxanthine in DMSO.

**Table S1.** End of reaction concentrations for Figure 2.

| OD_600_ | Starting Caffeine Concentration (mM) | Caffeine Consumed (μM) | 7-Methylxanthine Produced (μM) |
| --- | --- | --- | --- |
| 50 | 5 | 3,381 ± 220 | 3,133 ± 275 |
| 25 | 5 | 1,724 ± 145 | 1,697± 55 |
| 20 | 2 | 1,353 ± 59 | 1,393 ± 48 |
| 10 | 2 | 945 ± 76 | 825 ± 65 |
| 10 | 1 | 854 ± 32 | 886 ± 14 |
| 5 | 1 | 420 ± 21 | 465 ± 3 |

**Table S2. Mass of Products Before and After HPLC Purification.**

|  | 7-Methylxanthine |
| --- | --- |
| Concentration-Derived Mass Pre-HPLC Purification (mg) | 183.8 |
| Concentration-Derived Mass Post-HPLC Purification (mg) | 171.5 |
| HPLC Purification Separation Efficiency (%) | 93.30 |
| Actual Mass Recovered (mg) | 153.3* |

* Some mass lost during collection of dried compounds due to adherence to the evaporation container.

**Supplemental Methods**

*Plasmid Construction*

The primers and templates used for each PCR reaction to generate inserts are given in Table S2. For plasmids pAD3, pBD3, pADP1, and pBDP1, the inserts contained two genes linked by the ribosomal binding site upstream of the first multiple cloning site from pACYCDuet-1 (pETrbs2, GAAGGAGATATACC). The pAD3 insert was generated by first generating AD5ʹ and AD3ʹ PCR fragments, which were then mixed in a 1:1 (v:v) ratio, followed by overlap extension PCR to generate a single bicistronic insert, AD3poly. Similarly, the pBD3, pADP1, and pBDP1 inserts were generated using overlap extension PCR to produce BD3poly, ADP1poly, and BDP1poly fragments, respectively. The pET32a(+) vector and inserts were digested by NdeI and BamHI, followed by ligation using T7 DNA ligase. Plasmid dDP1DP1 was generated by inserting single *ndmDP1* genes into the NcoI/BamHI and NdeI/KpnI restriction sites.

**Table S3.**

| **Fragment** | **Template** | **Forward Primer** | **Reverse Primer** |
| --- | --- | --- | --- |
| AD5ʹ | dDA | ndmA-F-NdeI | ADP1poly-NR |
| AD3ʹ | dDB | ADP1poly-CF | ndmD-R-BamHI |
| AD3poly | AD5ʹ + AD3ʹ | ndmA-F-NdeI | ndmD-R-BamHI |
| BD5ʹ | dDB | ndmB-F-NdeI | BDP1poly-NR |
| BD3ʹ | dDA | BDP1poly-CF | ndmD-R-BamHI |
| BD3poly | BD5ʹ + BD3ʹ | ndmB-F-NdeI | ndmD-R-BamHI |
| ADP13ʹ | dDP1DP1 | ADP1poly-CF | ndmD-R-BamHI |
| ADP1poly | AD5ʹ + ADP13ʹ | ndmA-F-NdeI | ndmD-R-BamHI |
| BDP13ʹ | dDP1DP1 | BDP1poly-CF | ndmD-R-BamHI |
| BDP1poly | BD5ʹ + BDP13ʹ | ndmB-F-NdeI | ndmD-R-BamHI |
| DP1-1 | dDD | DP1-F-NcoI | ndmD-R-BamHI |
| DP1-2 | dDD | DP1-F-NdeI | ndmD-R-KpnI |

**Table S4.** Primers used in plasmid construction

| **Name** | **Primer Sequence (5’➝ 3’)** |
| --- | --- |
| ndmA-F-NdeI | GCACGGCAT**ATG**GAGCAGGCGATCATCAATGATGA |
| ndmB-F-NdeI | GCAAGGTCAT**ATG**AAAGAACAGCTCAAGCCGCTGC |
| ndmD-R-BamHI | GGGACGGGGATCC**TCA**CAGATCGAGAACGATTTTTTTGGA |
| ADP1poly-NR | CATGGTATATCTCCTTCTTATATGTAGCTCCTATCGC |
| BDP1poly-NR | CATGGTATATCTCCTTCTTACTGTTCTTCTTCAATAAC |
| DP1-F-NdeI | GCACTGCAT**ATG**ACTAAGGCTCCTCCAAC |
| ndmD-R-KpnI | GGGACGGGGTACC**TCA**CAGATCGAGAACGATTTTTTTGGA |
| DP1-F-NcoI | GTAAGATCC**ATG**GCTAAGGCTCCTCCAAC |
| ADP1poly-CF | GCGATAGGAGCTACATATAAGAAGGAGATATACCATG |
| BDP1poly-CF | GTTATTGAAGAAGAACAGTAAGAAGGAGATATACCATG |
